# Expression of the immune targets in tumor-infiltrating immunocytes of gestational trophoblastic neoplasia

**DOI:** 10.3389/pore.2023.1610918

**Published:** 2023-02-16

**Authors:** Hongyan Cheng, Liju Zong, Shuangni Yu, Jie Chen, Xirun Wan, Yang Xiang, Junjun Yang

**Affiliations:** ^1^ Department of Obstetrics and Gynecology, National Clinical Research Centre for Obstetric and Gynecologic Diseases, Peking Union Medical College Hospital, Chinese Academy of Medical Sciences and Peking Union Medical College, Beijing, China; ^2^ Department of Pathology, Peking Union Medical College Hospital, Chinese Academy of Medical Sciences and Peking Union Medical College, Beijing, China

**Keywords:** gestational trophoblastic neoplasia, tumor-infiltrating immunocyte, PD-1, TIM-3, GAL-9, LAG-3

## Abstract

**Objectives:** To evaluate the expression of emerging immune targets in the tumor-infiltrating immunocytes (TIIs) of human gestational trophoblastic neoplasia (GTN) specimens, and to analyze the correlation between the expression patterns and prognosis of GTN patients.

**Methods:** Between January 2008 and December 2017, patients who were diagnosed histologically with GTN were included in this study. The expression densities of LAG-3, TIM-3, GAL-9, PD-1, CD68, CD8, and FOXP3 in the TIIs were assessed independently by two pathologists blinded to clinical outcomes. The expression patterns and correlation with patient outcomes were analyzed to identify prognostic factors.

**Results:** We identified 108 patients with GTN, including 67 with choriocarcinoma, 32 with placental site trophoblastic tumor (PSTT), and 9 with epithelioid trophoblastic tumor (ETT). Almost all GTN patients showed expression of GAL-9, TIM-3, and PD-1 in TIIs (100%, 92.6%, and 90.7%, respectively); LAG-3 was expressed in 77.8% of the samples. The expression densities of CD68 and GAL-9 were significantly higher in choriocarcinoma than that in PSTT and ETT. The TIM-3 expression density in choriocarcinoma was higher than that in PSTT. In addition, the expression density of LAG-3 in the TIIs of choriocarcinoma and PSTT was higher than that in ETT. There was no statistical difference in the expression pattern of PD-1 among different pathological subtypes. The positive expression of LAG-3 in tumor TIIs was a prognostic factor for disease recurrence, and patients with positive expression of LAG-3 in the TIIs had poorer disease-free survival (*p* = 0.026).

**Conclusion:** Our study evaluated the expression of immune targets PD-1, TIM-3, LAG-3, and GAL-9 in the TIIs of GTN patients and found that they were widely expressed but not associated with patients’ prognoses, excepting the positive expression of LAG-3 was a prognostic factor for disease recurrence.

## Introduction

Gestational trophoblastic neoplasia (GTN) is a group of malignant diseases arising from the abnormal proliferation of placental trophoblastic cells, which includes invasive mole, choriocarcinoma, placental site trophoblastic tumor (PSTT), and epithelioid trophoblastic tumor (ETT) (1). The specific marker beta-human chorionic gonadotropin (β-HCG) is secreted by tumor cells and can be used as a monitoring index for treatment. Although almost all low-risk patients can be cured with standardized chemotherapy, the five-year survival rate is less than 70% for patients with drug-resistant and recurrent disease (2, 3). Recently, emerging immunotherapies such as programmed cell death 1 (PD-1) inhibitors have shown excellent efficacy in the treatment of drug-resistant and recurrent GTN (4).

The fetus and fetus-derived trophoblast cells are part of the semi-allogeneic conceptus; however, the fetus and placenta are not rejected by the maternal immune system because the fetus and its affiliated tissues express paternal antigens that establish immune tolerance at the maternal–fetal interface (5). Previous studies have shown that programmed cell death ligand (PD-L1) is expressed in human placentas and GTN specimens (6,7,8). The B7 family and their receptors are major immune checkpoints that regulate the activation and function of T cells. In addition to PD-L1, other immunoregulatory molecules of the B7 family show high expression in GTN, including B7-H3 and the V-type immunoglobulin domain–containing suppressor of T cell activation (8).

In addition to T cells, tumor-infiltrating immunocytes (TIIs) also include macrophages, natural killer (NK) cells, and dendritic cells (DC). Many studies have investigated the expression of immune checkpoints in tumor cells, but there is no study on these in TIIs of GTN. In this study, we investigated the expression of PD-1, lymphocyte activation gene-3 (LAG-3), T cell immunoglobulin-3 (TIM-3), and galectin-9 (GAL-9) in the TIIs of human GTN specimens and analyzed the correlation between expression difference and prognosis of GTN patients to explore potential new targets for immunotherapy of drug-resistant and recurrent GTN.

## Materials and Methods

### Patients and samples

This study included patients diagnosed histologically with choriocarcinoma, PSTT, or ETT and who had enough tumor samples for immunohistochemistry at Peking Union Medical College Hospital (Beijing, China) between January 2008 and December 2017. The clinicopathological data were obtained from patients’ admission and discharge files. The treatment and follow-up methods have been described as our previous studies (3, 8, 9). This study was approved by the Institutional Review Board (SK-995); informed consent was not required due to its retrospective nature.

### Immunohistochemistry and assessment

Immunohistochemistry was performed using our laboratory protocol described previously (8,9). Briefly, 4-μm serial sections were deparaffinized and subjected to heat-induced epitope retrieval using 10 mM sodium citrate (pH 6.0) at 95°C for 20 min. The endogenous peroxidase activity was quenched using a 0.3% hydrogen peroxide solution. Sections were incubated with primary antibodies against LAG-3 (Clone D4G40, 1:100), TIM-3 (Clone D5D5R, 1:200), GAL-9 (Clone D9G40, 1:200), PD-1 (Clone D4W2J, 1:200), CD68 (Clone D4B9C, 1:500), CD8 (Clone D8A8Y, 1:200), and FOXP3 (Clone D2W8E, 1:200). All antibodies were obtained from Cell Signaling Technology (Boston, United states). All slides were stained using an automatic immunohistochemistry staining instrument (Bond Max, Leica Biosystems; Buffalo Grove, IL, United states) according to the manufacturer’s protocol. Human tonsil tissues stained using the primary antibodies were used as positive controls; the same tissues with isotypematched immunoglobulins comprised the negative controls.

The tumors were distinguished from the stroma and TIIs using hematoxylin and eosin staining. The expression of LAG-3, TIM-3, GAL-9, PD-1, CD68, CD8, and FOXP3 in the TIIs was assessed independently by two pathologists (LJ. Z and SN. Y), who were blinded to clinical outcomes. Five representative fields were selected for each slide at ×40 magnification. The percentages of cells that stained positive for each marker were quantified in 5% increments of the overall tumor section. The density of the positive immune cells in the TIIs was classified using a semi-quantitative score as 0 (negative, <5%), 1 (sporadic, 5%–25%), 2 (moderate, 25%–50%), or 3 (strong positive, >50%).

### Statistical analysis

The χ^2^ test or Fisher’s exact test was used to evaluate the association between categorical variables. The Kruskal–Wallis (H) test and Dunn *post hoc* test were performed to evaluate expression differences among the different pathological types of GTN. Disease-free survival (DFS) was defined as the period between surgery and the detection of the first local, regional, or distant relapse. Overall survival (OS) was defined as the period between surgery and death from any cause. The Kaplan-Meier survival analysis and log-rank test were performed to describe recurrence and OS. All statistical analyses were performed using SPSS software version 19 (IBM Company, Armonk, NY, United states). A two-sided *p*-value < 0.05 (or a Bonferroni-corrected significance threshold) was considered statistically significant.

## Results

### Clinical characteristics

A total of 108 patients had adequate tumor samples for immunohistochemistry; 67 were diagnosed with choriocarcinoma, 32 had PSTT, and 9 had ETT. The median age at surgery was 32 (range: 21–53) years. The prognostic score according the 2000 International Federation of Gynecology Obstetrics (FIGO) scoring system, had a median of 8 (range: 1–15 scores) (10). Of the 67 choriocarcinoma specimens, 7 were from hysterectomies or diagnostic curettages, 57 from pulmonary metastases, 2 from brain metastasis, and 1 from intestinal metastasis. Among the 108 samples, 34 were from chemo-naive patients, and 74 were obtained from patients after chemotherapy (57 relapsed and 17 chemo-refractory). All PSTT and ETT specimens were acquired *via* hysterectomy, except in one ETT case (recto-uterine pouch), as reported previously (11). The detailed clinicopathological characteristics of the patients are shown in Table 1.

**TABLE 1:** Clinicopathologic characteristics of patients with gestational trophoblastic neoplasia (*n* = 108).

Characteristics	N (%)
Pathological type	
Choriocarcinoma	67 (62.1)
PSTT	32 (29.6)
ETT	9 (8.3)
Disease type	
Chemo-naive	34 (31.5)
Relapsed	57 (52.8)
Chemo-refractory	17 (15.7)
Age	
<40	91 (84.3)
≥40	17 (15.7)
FIGO stage	
I	29 (26.8)
II	3 (2.8)
III	64 (59.3)
IV	12 (11.1)
FIGO score	
1–3	27 (25.0)
4–6	11 (10.2)
7–12	62 (57.4)
12	8 (7.4)
Specimen type	
Uterus	47 (43.5)
Pulmonary metastasis	57 (52.8)
Brain metastasis	2 (1.9)
Recto-uterine pouch	1 (0.9)
Intestinal metastasis	1 (0.9)

### Expression of immune targets

**FIGURE 1:**
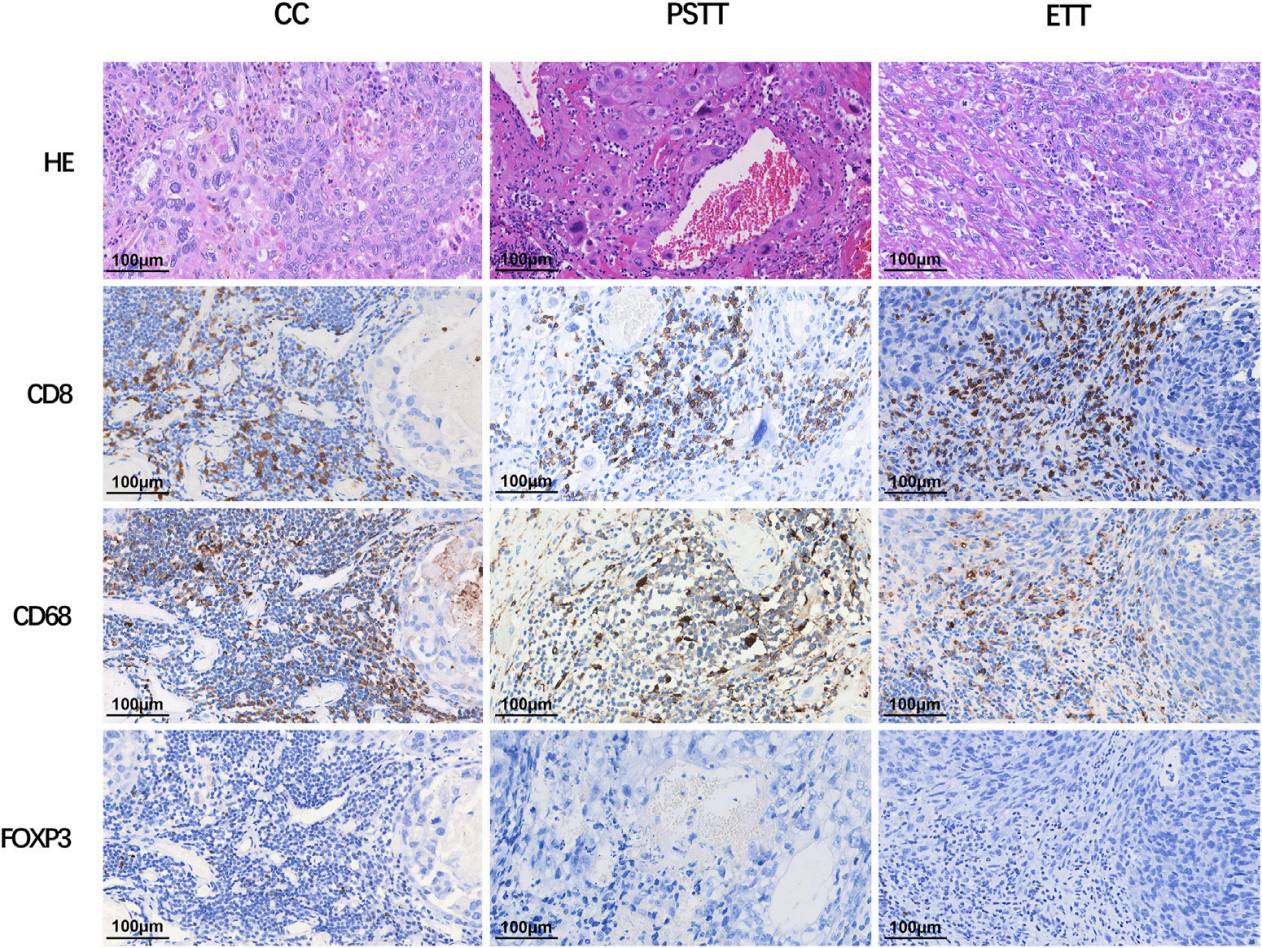
Expression of CD8, CD68, and FOXP3 in different types of gestational trophoblastic neoplasia. CC: choriocarcinoma; PSTT: placental site trophoblastic tumor; ETT: epithelioid trophoblastic tumor; HE: hematoxylin and eosin staining.

**FIGURE 2:**
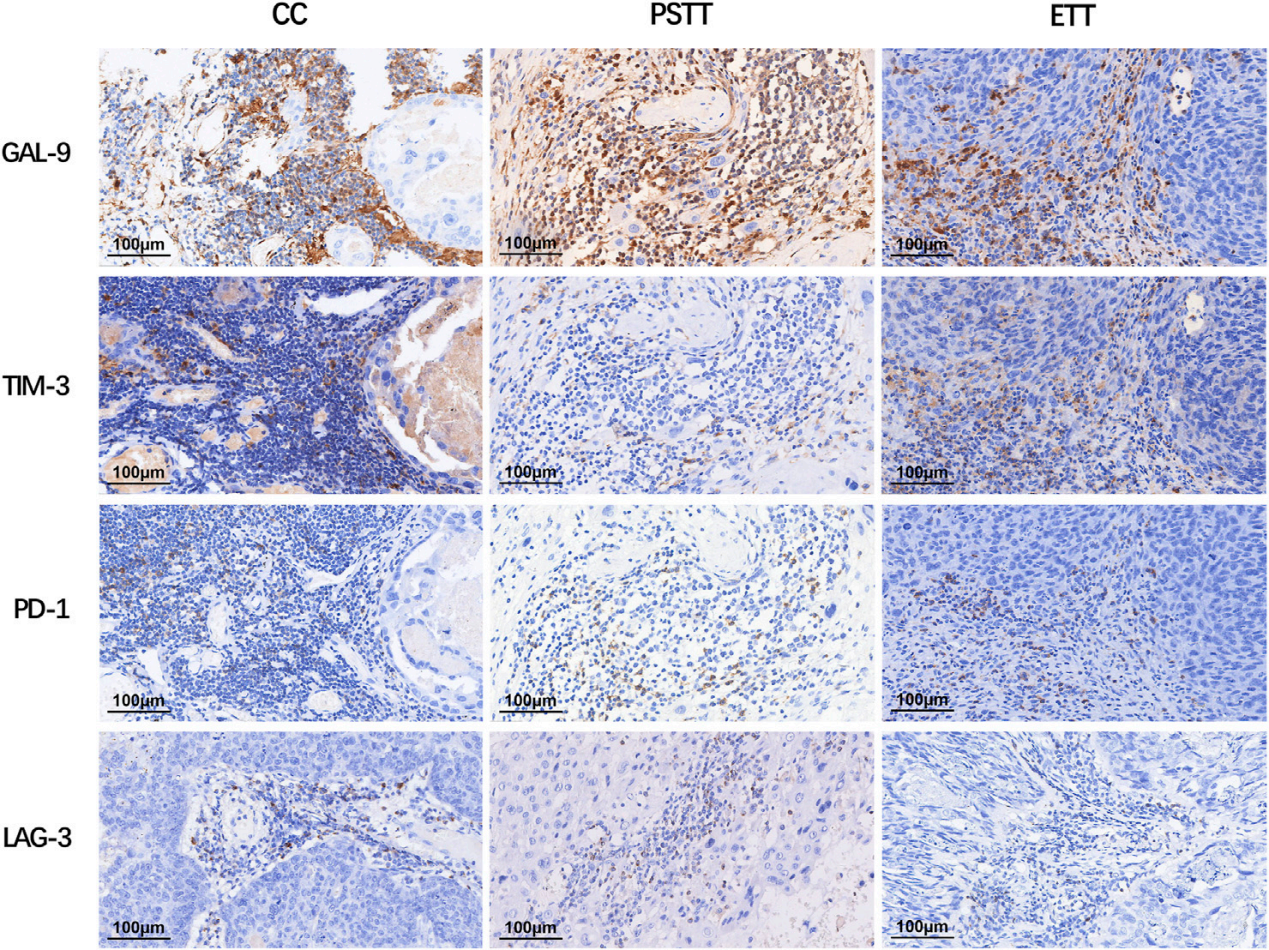
Expression of immune targets in tumor-infiltrating immunocytes of gestational trophoblastic neoplasia. LAG-3: lymphocyte activation gene-3; TIM-3: T cell immunoglobulin-3; GAL-9: galectin-9; PD-1: programmed cell death 1.

As shown in Figure 1, CD8^+^ T cells and CD68^+^ macrophages were widely infiltrated in all GTN stromal cells (100% and 98.1%, respectively). FOXP3^+^ regulatory T cells (Tregs) were detected in 8 choriocarcinoma samples but were not detected in PSTT and ETT. The expression of PD-1 in the TIIs was positive in 98 (90.7%) patients, and moderate-to-strong density was detected in 56 patients (51.9%) (Figure 2). TIM-3 and GAL-9 were expressed in the TIIs of almost all GTN patients (92.6% and 100%, respectively). A sporadic density for TIM-3 was noted in 55 patients (50.9%), and moderate -to-strong density was found in 45 patients (41.7%). In contrast to TIM-3, 102 patients (94.4%) were found to have a moderate-to-strong density of GAL-9 expression. LAG-3 showed positive expression in 84 patients (77.8%), 79 of which had sporadic expression, 4 had moderate expression, and one was strongly positive. The expression of each immune target in GTN is shown in Table 2.

**TABLE 2:** Expression of immunomarkers of tumor-infiltrating immunocytes in gestational trophoblastic neoplasia (n = 108).

	LAG-3	TIM-3	GAL-9	PD-1	CD68	CD8	FOXP3
Density of positive TIIs							
0	24	8	0	10	2	0	100
1	79	55	6	42	34	25	8
2	4	35	27	50	42	64	0
3	1	10	75	6	30	19	0

### Expression patterns and correlations among pathological subtypes of gestational trophoblastic neoplasia

**FIGURE 3:**
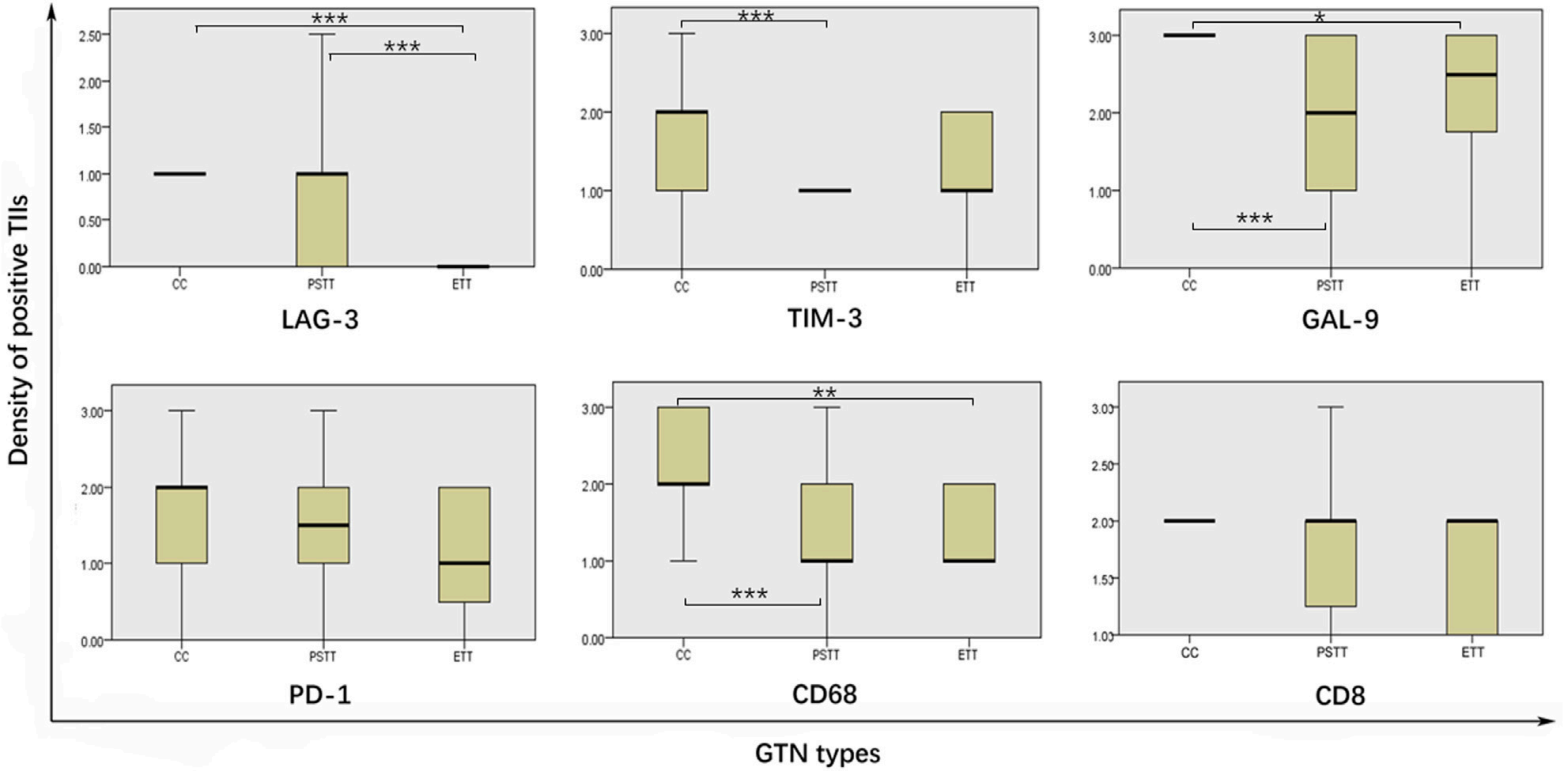
Comparison of immune targets expression level in different pathological gestational trophoblastic neoplasia types. TIIs: tumor-infiltrating immunocytes; GTN: gestational trophoblastic neoplasia; * = *p* < 0.01, ** = *p* < 0.01, *** = *p* < 0.001.

The expression differences of LAG-3, TIM-3, GAL-9, PD-1, CD68, and CD8 in TIIs among three pathological subtypes are shown in Table 3 and Figure 3. The Kruskal–Wallis test results showed that the expression density of CD68 and GAL-9 in choriocarcinoma TIIs was significantly higher than that in PSTT and ETT. The expression density of TIM-3 in choriocarcinoma TIIs was higher than that in PSTT. Moreover, the expression of LAG-3 in the TIIs of choriocarcinoma and PSTT was higher than that in ETT. There was no statistical difference in the expression pattern of PD-1 across pathological subtypes. As shown in Supplementary Tables S1, S2, statistical testing showed that LAG-3 expression was associated with the expression of TIM-3, PD-1, CD68, and FOXP3, and PD-1 expression was found associated with TIM-3 and CD68. The association between the expression patterns of these targets suggests a positive correlation and co-expression.

**TABLE 3:** Expression of immunomarkers of tumor-infiltrating immunocytes in different pathological subtypes of gestational trophoblastic neoplasia.

Targets	Density	Choriocarcinoma	PSTT	ETT	*p*
LAG-3	0	7	9	8	<0.001*
	1	57	21	1	
	2	3	1	0	
	3	0	1	0	
TIM-3	0	1	6	1	<0.001*
	1	28	22	5	
	2	28	4	3	
	3	10	0	0	
GAL-9	0	0	0	0	<0.001*
	1	0	5	1	
	2	7	17	3	
	3	60	10	5	
PD-1	0	2	6	2	0.581
	1	29	10	3	
	2	34	12	4	
	3	2	4	0	
CD68	0	0	2	0	<0.001*
	1	9	20	5	
	2	30	8	4	
	3	28	2	0	
CD8	0	0	0	0	0.145
	1	13	8	4	
	2	41	18	5	
	3	13	6	0	
FOXP3	0	59	32	9	0.073
	1	8	0	0	
	2	0	0	0	
	3	0	0	0	

### Survival outcome

**FIGURE 4:**
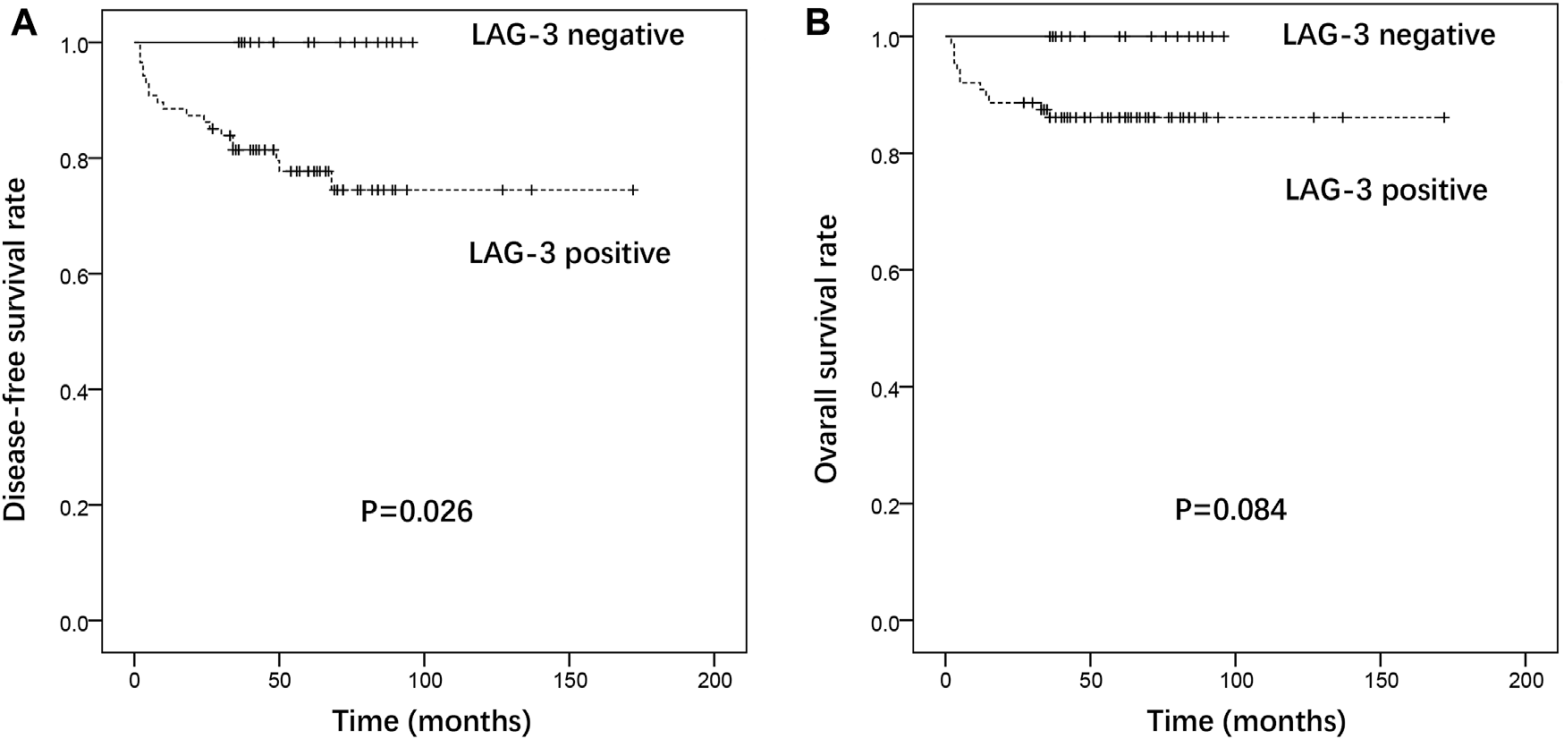
Association of LAG-3 expression with disease-free survival **(A)** and overall survival **(B)** in gestational trophoblastic neoplasia.

At the last follow-up, 12 patients had died from GTN, 7 were alive with GTN, and 89 had achieved complete remission. The follow-up duration among the 96 patients alive at their last follow-up had a median of 60 months (range: 27–172 months). The positive expression of LAG-3 in tumor TIIs was a prognostic factor for disease recurrence in patients, and patients with positive expression of LAG-3 in TIIs had a poorer DFS (*p* = 0.026). Patients with negative expression of LAG-3 had higher OS, but there was no statistical difference (3-year OS: 100% vs 81.4%, 5-year OS: 100% vs 77.7%) (Figure 4). There was no association between patients’ outcomes and the expression of other immune targets in the TIIs.

## Discussion

In the present study, we found that the immune targets PD-1, TIM-3, LAG-3, and GAL-9 were widely expressed in the TIIs of GTN patients but not associated with the patients’ prognoses, excepting that the positive expression of LAG-3 was a prognostic factor for disease recurrence. The co-expressions among these immune targets may support treatments using immune checkpoint inhibitor combinatorial approaches. The co-expression of multiple immune molecules was not prognostic, consistent with previous reports in ovarian cancer and lung cancer (12, 13).

The correlation between the origins of GTN tumors and immune tolerance suggests that immunotherapy may be a promising salvage treatment for drug-resistant and recurrent GTN. Research focused on PD-L1 expression in GTN tumor cells showed that inhibitors targeting PD-1 in GTN were therapeutically efficacious in several cases (4,6,7,8). Although our study showed that PD-1^+^ TIIs were present in 90.7% of GTN patients, its expression does not affect a patient’s outcome. It has been reported that PD-1^+^ CD4^+^ tumor infiltrating lymphocytes (TILs) and CD8^+^ TILs had less proliferative capacity comparing to the PD-1^-^ CD8^+^ or CD4^+^ TILs, suggesting that both CD8^+^ and CD4^+^ TILs are functionally anergic through activation of the PD-1/PD-L1 pathway (14). Notably, multiple clinical trials are currently investigating the efficacy of PD-1/PD-L1 inhibitors in GTN patients (NCT: 03135769 and 04047017).

LAG-3 structurally resembles the CD4 co-receptor and is expressed mainly on activated T cells, DCs, and NK cells (15). LAG-3 binds the class II major histocompatibility complex (MHC II) with a high affinity. Studies indicated that the blockade of LAG-3 improves cytotoxic T lymphocyte proliferation and effector function (16, 17). The frequency of antigenic sharing for the human leukocyte antigen (HLA) DR locus in GTN couples is significantly higher than that in normal fertile couples, indicating that the states of an MHC-linked gene (HLA-DR) influence the development of GTN (18). LAG-3 may play an important role in the development of ovarian and cervical cancer (19, 20). Dual blockade of LAG-3 and PD-1 efficiently augments proliferation and cytokine production by CD8^+^ T cells (21). This study is the first to identify that LAG-3 is expressed in the majority of GTN patients. In addition, the positive expression of LAG-3 in tumor TIIs was a prognostic factor for disease recurrence and poorer DFS in patients with GTN. Patients with negative expression of LAG-3 had a higher OS rate; however, it was not statistically different. Since the expression pattern in different pathological types was not significant, these results suggest that the expression of LAG-3 in tumor TIIs may be a prognostic factor and therapeutic target in GTN.

TIM-3 is a type I transmembrane protein expressed mainly on CD8^+^ and CD4^+^ T cells, DCs, monocytes, NK cells, and Tregs (22). GAL-9 is a ligand of TIM-3. The binding of TIM-3 with GAL-9 promotes calcium influx, cell aggregation, and apoptosis, further inhibiting the activation and proliferation of T cells and ultimately inducing peripheral immune tolerance (23, 24). In this study, TIM-3 and GAL-9 were expressed in the TIIs of almost all GTN patients. Moderate -to-strong densities of TIM-3 and GAL-9 were found in 41.7% and 94.4% of patients, respectively. Although studies have shown that high expression of GAL-9 in solid tumors is associated with a poor prognosis (25), our data did not show a correlation between GAL-9 expression in TIIs and patient outcomes. Additionally, the effects of the TIM-3/GAL-9 pathway on innate immunity are different from those of adaptive immunity. In the study of Lv et al. (26), overexpression of GAL-9 was associated with M2 macrophages, whereas its downregulation promoted macrophage polarization into M1macrophages. A previous study had indicated that M1 macrophages greatly enhance cytotoxicity and reduce tumor growth, whereas M2 macrophages increase cell invasion and survival (27).

Our results showed that macrophages were detected constitutively in almost all GTNs; their density in choriocarcinoma was significantly higher than those in PSTT and ETT but unrelated to patient outcomes. Macrophages are traditionally considered as typical antitumor cell in innate immunity, and tumor-associated macrophages can directly kill cancer cells and stimulate the antitumor activity of T cells. However, clinical and basic studies have shown that tumor-associated macrophages can promote the malignant progression of tumors in some cases (28-30). Moreover, studies have suggested that there is a strong association between poor survival and increased macrophage density in solid tumors (28, 29). . The tumor-promoting functions of macrophages include tumor angiogenesis, cell invasion, migration, and intravasation, as well as the inhibition of antitumor immune responses (28, 30). In addition to GAL-9 inducing polarization of macrophages from the M1 to M2 type, as mentioned above, tumor-associated macrophages can also inhibit T cell function through the PD-1/PD-L1 axis (31). In summary, therapies targeting tumor-associated macrophages may complement the immunosuppressive effects of the PD-1/PD-L1 pathway.

This study had some limitations. First, selection bias may have occurred due to the retrospective nature of the study. Second, atypical necrosis of tumor tissue after chemotherapy adds difficulty to the interpretation of pathological findings. Third, the heterogenous set of samples from different subtypes may have led to the lack of distinction in immune marker expression. In addition, immunohistochemical methods cannot distinguish the expression of targets among immunocyte subtypes.

## Conclusion

Evaluation of the expression of immune targetsPD-1, TIM-3, LAG-3, and GAL-9 in GTN TIIs showed that they were widely expressed in TIIs but not associated with patients’ prognoses, excepting the positive expression of LAG-3 was a prognostic factor for disease recurrence. These findings suggest that LAG-3 may provide the basis for the study of pathogenesis and be a novel therapeutic target in GTN treatment.

## Data Availability

The raw data supporting the conclusion of this article will be made available by the authors, without undue reservation.
