# Supplementary material for: Expression of the immune targets in tumor-infiltrating immunocytes of gestational trophoblastic neoplasia
Source: Pathol Oncol Res. 2023 Feb 16;29:1610918. doi: 10.3389/pore.2023.1610918 (PMC9977799; doi:10.3389/pore.2023.1610918)
Supplement: Supplementary file 1 [file DataSheet1.docx]

**Supplementary Table S1.** Expression correlation between LAG-3 and other target (Fisher exact test or χ^2^ test).

| Targets | | | | *P* value |
| --- | --- | --- | --- | --- |
| LAG-3 and TIM-3 | | | | 0.013 |
|  |  | TIM-3 | |  |
|  |  | Positive | Negative |  |
| LAG-3 | Positive | 81 | 3 |  |
|  | Negative | 19 | 5 |  |
| LAG-3 and PD-1 | | | | 0.008 |
|  |  | PD-1 | |  |
|  |  | Positive | Negative |  |
| LAG-3 | Positive | 80 | 4 |  |
|  | Negative | 18 | 6 |  |
| LAG-3 and FOXP3 | | | | 0.013 |
|  |  | FOXP3 | |  |
|  |  | Positive | Negative |  |
| LAG-3 | Positive | 3 | 81 |  |
|  | Negative | 5 | 19 |  |
| LAG-3 and CD68 | | | | 0.048 |
|  |  | CD68 | |  |
|  |  | Positive | Negative |  |
| LAG-3 | Positive | 84 | 0 |  |
|  | Negative | 22 | 2 |  |

**Supplementary Table S2.** Expression correlation between PD-1 and other target (Fisher exact test or χ^2^ test).

| Targets | | | | *P* value |
| --- | --- | --- | --- | --- |
| PD-1 and TIM-3 | | | | 0.025 |
|  |  | TIM-3 | |  |
|  |  | Positive | Negative |  |
| PD-1 | Positive | 93 | 5 |  |
|  | Negative | 7 | 3 |  |
| PD-1 and CD68 | | | | 0.008 |
|  |  | CD68 | |  |
|  |  | Positive | Negative |  |
| PD-1 | Positive | 98 | 0 |  |
|  | Negative | 8 | 2 |  |
